# Short-term pectin-enriched smoothie consumption has beneficial effects on the gut microbiota of low-fiber consumers

**DOI:** 10.1093/femsmc/xtae001

**Published:** 2024-02-02

**Authors:** Susan Pihelgas, Kristel Ehala-Aleksejev, Rain Kuldjärv, Ann Jõeleht, Jekaterina Kazantseva, Kaarel Adamberg

**Affiliations:** TFTAK, Mäealuse 2/4B, 12618 Tallinn, Estonia; School of Natural Sciences and Health, Tallinn University, Narva mnt 25, 10120 Tallinn, Estonia; TFTAK, Mäealuse 2/4B, 12618 Tallinn, Estonia; TFTAK, Mäealuse 2/4B, 12618 Tallinn, Estonia; TFTAK, Mäealuse 2/4B, 12618 Tallinn, Estonia; TFTAK, Mäealuse 2/4B, 12618 Tallinn, Estonia; TFTAK, Mäealuse 2/4B, 12618 Tallinn, Estonia; Department of Chemistry and Biotechnology, Tallinn University of Technology (TalTech), Akadeemia tee 15, 12618 Tallinn, Estonia

**Keywords:** dietary fibre, pectin, gut microbiota, intervention trial, 16S rRNA sequencing

## Abstract

Adequate consumption of fiber has a positive effect on health. The crossover study examined the effect of a pectin-enriched smoothie on gut microbiota and health parameters. During 3 weeks, 31 adults consumed two smoothies (11.6 or 4.8 g of fiber/day), alternating with washout periods in different order. At the end of each period, weekly food diaries, blood samples, and stool microbiota were collected. Changes in the microbiota during smoothie consumption were associated with baseline fiber intake. A greater proportion of up- (*Lachnospira, Colidextribacter*, and *Bacteroides*) or down-shifts (*Streptococcus, Holdemanella*) was observed in low-fiber (*n* = 22) compared to high-fiber consumers (*n* = 9). In both groups, the pectin-enriched smoothie reduced the number of the *Ruminococcus torques group* bacteria. Our results showed that the short-term approach is effective to estimate relationships between food components and gut bacteria.

## Introduction

Human gut microbiota consists of about 10^13^ microbial cells that inhabit mainly in the large intestine (Sender et al. 2016a,b). The diversity and richness of gut microbiota have a remarkable impact on human health through the production of different metabolites, vitamins, amino acids, and signaling molecules. The composition of microbiota is affected to a large extent by host genotype, diseases, environmental changes, and diet (Thursby and Juge 2017). It is supposed that even a very short-term, altered diet can modulate the human gut microbiome rapidly. For example, *Prevotella* abundance decreases in vegetarian subjects during a five-day long animal-based diet (David et al. 2014). However, it seems that the animal-based diet has a greater impact on the gut microbiota than the plant-based diet. It has been described that an animal-based diet increases bile acid-tolerant microorganisms like *Alistipes, Bilophila*, and *Bacteroides* and decreases the abundance of bacteria that metabolize dietary plant polysaccharides—*Roseburia, Eubacterium rectale*, and *Ruminococcus bromii* (David et al. 2014).

Dietary fiber (DF) is a type of carbohydrate that is mostly not digestible by enzymes produced by humans. DF is transported through the digestive system to the large intestine, where it is metabolized by gut microbiota. The most abundant products of DF metabolism are short-chain fatty acids (SCFA) like butyrate, acetate, and propionate (Cummings 1981). These SCFAs differ in their fate and tissue distribution and can affect lipid, glucose, and cholesterol metabolism (Den Besten et al. 2013, Chambers et al. 2018).

DF can be divided into different groups—non-digestible oligosaccharides (inulin), non-starch polysaccharides (pectin, beta-glucan), and resistant starch (Cummings and Macfarlane 1991). Pectin is one of the DFs that has several beneficial effects on human health, including reduced glucose and cholesterol absorption (Stephen et al. 2017, Salleh et al. 2019); an increase of fecal mass (Cummings et al. 1978); and as a substrate for gut bacteria (Blanco-Pérez et al. 2021). Thus, citrus pectin oligosaccharides have been shown to have a modulating effect on cholesterol metabolism involving specific groups of bacteria along with their metabolites (Hu et al. 2019). Beta-glucan, known for its ability to enhance bile acid production from cholesterol in the liver and promote its elevated excretion in the feces, is also linked to beneficial health effects. It has been reported that beta-glucan supplementation causes reductions in cholesterol and triglycerides (Cronin et al. 2021). Also, some studies show that psyllium may improve the lipid profile (Deng et al. 2022).

Consuming a diverse range of fiber-rich foods is frequently linked to enhanced bowel movement, lower body mass index (BMI), and improved glucose and cholesterol levels, all of which are correlated with a decreased risk of cardiovascular disease, obesity, and type 2 diabetes (Reynolds et al. 2019). A systematic review and meta-analysis of 12 randomized controlled trials suggests that supplementation with isolated soluble fiber can improve body composition and metabolic outcomes, including fasting glucose and insulin levels (Thompson et al. 2017). The recommended daily fiber intake depends on daily energy consumption and is about 13 g per 1000 kcal (Pitsi et al. 2017, Stephen et al. 2017). However, the real amount of DF consumption is usually much lower for people following a Western-type diet enriched with products of animal origin (Stephen et al. 2017).

To date, a complex approach that includes keeping a food diary, measuring general body parameters, conducting extensive blood tests, and continuously characterizing gut microbiota in relation to two different levels of increased fiber consumption and following washing-time periods has not been performed. The present study aimed to evaluate the effect of a smoothie enriched with pectin and other soluble fibers on human gut microbiota and health parameters depending on the daily fiber intake during a short-term intervention study. The results of this study provide new insight into the support of beneficial bacteria by increased intake of soluble DFs.

## Materials and methods

The current study was carried out and the smoothies were developed by the Center of Food and Fermentation Technologies (TFTAK, Estonia) in collaboration with Siidrikoda (Estonia).

### Composition of the smoothies

In the present study, we used two different fruit smoothies with distinctive fiber (esp. pectin) content. The smoothies consisted mainly of fruit purees and concentrates. The high-pectin (HPect) smoothie recipe was developed with the aim of utilizing apple pomace from the cider production facility Siidrikoda, thereby adding value to the byproduct. It contained 36% of apple puree and a special dietary supplement included pectin, psyllium, and beta-glucan fibers (Calm your rumbly tummy, Elsavie, Estonia). The low-pectin smoothie (LPect) contained an appropriate amount of starch. The exact recipes of the smoothies are presented in the Supplementary Table S1, and the nutritional values of the smoothies per 100 g are shown in the Supplementary Table S2.

### Recruitment of study participants and design of the study

The current study was carried out from October 2022 to December 2022. Participants were selected according to a questionnaire and definite criteria. The questions concerned the respondents’ eating habits, description of their health status (incl. digestive health), and data on their general lifestyle. The target group of the study included individuals who met at least 3 of the following conditions: sedentary lifestyle, low Bristol score (<3), modest consumption of fruits and vegetables, and gastrointestinal complaints without a diagnosed disease. The exclusion criteria were as follows: antibiotic use 3 months before the study, any severe or chronic diseases (e.g. cancer, Crohn’s disease, ulcerative colitis, etc.), specific diet (e.g. ketogenic, vegan, low carbohydrate, high-fat diet), travel to subtropic or tropic area within 3 months, or intolerance of apples. Participants were asked to follow their usual eating habits throughout the study. Subjects who did not follow the study protocol were excluded from the study. Out of 368 volunteers who completed the questionnaire, 55 were men, representing 15% of the total participants. Out of the 39 eligible participants, 31 completed the study, of which 3 (9.7%) were men.

**Figure 1.**
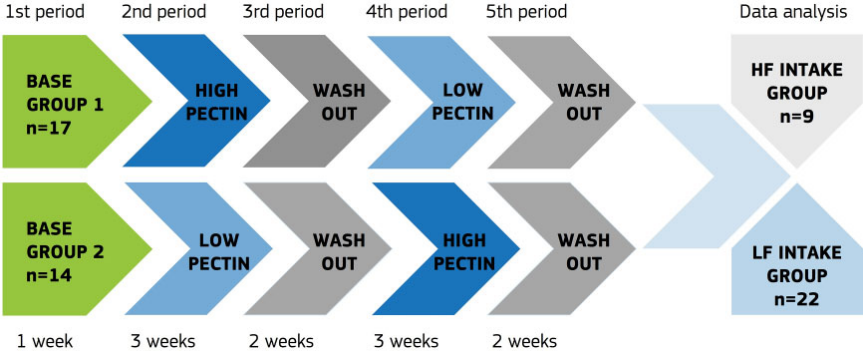
The study was divided into 5 periods: a base period to describe the individual health parameters and food habits at a starting point, two different smoothie consumption periods (with high and low pectin content), followed by smoothie WO periods. WO periods prefigure a regular menu and no smoothie consumption; after that, participants were grouped based on their daily fiber intake. Low-fiber intake group (LF) consumed <23 g of DF per day (*n* = 22), high-fiber intake group (HF) consumed >23 g of fiber per day (*n* = 9).

The study consisted of five periods (Fig. 1). The one-week base period was followed by two three-week test periods with alternating two-week washout (WO) periods. Test periods encompass two different smoothie consumption (one per period) in addition to the regular menu.

Following the base period, subjects were randomly divided into two groups based on the first smoothie they ate, while the study participants were unaware of the type of smoothie they were consuming. Group 1 (*n* = 17) consumed a HPect smoothie first, and group 2 (*n* = 14) started with an LPect smoothie. After the WO period, the smoothies were switched between the groups (Fig. 1).

For data analysis, subjects were grouped into low-fiber (LF) and high-fiber (HF) intake cohorts based on the analysis of food diaries according to the Estonian nutrition and movement recommendations (Pitsi et al. 2017) and their everyday DF consumption (during base and WO periods). The LF intake group (*n* = 22) regularly consumed <23 g of DF per day, while the HF intake group (*n* = 9) consumed >23 g of DF per day (Fig. 1). All participants signed written informed consent forms before the beginning of the study. The research was approved by the local ethics committee (Research Ethics Committee of the National Institute for Health Development, Reference number 1065, issued on 04/25/2022).

### Data collection

Nutrition data diaries were filled out one week before blood and fecal sample collection using the NutriData dietary analysis program (National Institute for Health Development, Estonia). The participants were asked to fill out the exact amount of smoothies they consumed. The analysis of macronutrients considered all regular diet periods (base and WO periods) to rule out occasional variations in the daily diet. At the end of every period, blood and fecal samples were taken (five blood and fecal samples per participant), and body composition was determined with a Tanita body composition analyzer (DC-360S, Tanita Corporation, Tokyo, Japan).

### Analyses of blood samples

Blood test analysis was performed by the Tartu University Hospital laboratory or SYNLAB Eesti OÜ (Tallinn, Estonia). Blood samples were taken between 7 a.m. and 10.30 a.m. after overnight fasting (on the same day when the fecal sample was produced). Blood was centrifuged and serum was used for analysis. The hexokinase method was used to determine glucose level. Enzymatic colorimetric method was used to measure blood lipids (total, LDL, HDL-cholesterol, and triglycerides), gamma glutamyl transferase (GGT), and uric acid levels (Cobas c 501, Roche Diagnostics). Serum alanine aminotransferase (ALAT) level was tested using a kinetic photometric method (Cobas 6000, Roche Diagnostics CH–6343 Rotkreuz, Switzerland). Immunoturbidimetric test was used to measure C-reactive protein level. An automatic analyzer was used to determine the hemogram together with the leukogram.

### DNA extraction and sequencing

Fecal samples were collected with DNA/RNA Shield Collection Tubes with Swabs (Zymo Research, Irvine, CA, USA) using FecesCatcher by TagHemi (Zeijen, The Netherlands) and stored at +4°C. Before DNA extraction, samples were frozen at −20°C at least overnight. DNA was extracted using the ZymoBIOMICS DNA Miniprep Kit (Zymo Research, Irvine, CA, USA) according to the manufacturer´s instructions. Qubit™ 3 Fluorometer (Thermo Fisher Scientific, Waltham, MA, USA) and the dsDNA BR Assay Kit (Thermo Fisher Scientific) were used for gDNA quantification.

The V4 hypervariable region of the 16S rRNA gene was PCR amplified using universal forward F515 5`-GTGCCAGCMGCCGCGGTAA-3` and reverse R806 5´-GGACTACHVGGGTWTCTAAT-3´ primers (Caporaso et al. 2011). Samples were sequenced using the Illumina MiSeq platform and a 2 × 150-cycles paired-end sequencing protocol. On average, 38 800 reads (minimum 17 677 reads) per sample were obtained. The whole sequencing workflow was published before (Kazantseva et al. 2021).

DNA sequence data was analyzed by BION-meta software (https://github.com/nielsl/mcdonald-et-al) according to the authors’ instructions (McDonald et al. 2016). The sequences were first cleaned at both ends using a 99.5% minimum quality threshold for at least 18 out of 20 bases for 5`-end and 28 out of 30 bases for 3`-end, followed by joining and removal of shorter contigs than 150 bp. Afterwards, the sequences were cleaned from chimeras and clustered by 95% oligonucleotide similarity (k-mer length of 8 bp, step size 2 bp). Finally, consensus reads were aligned to the SILVA reference 16S rRNA database (v138) using a word length of 8 and a similarity cut-off of 90%.

### Statistical analyses

Statistical analyses were performed at the bacteria genus level with abundance at least >0.0006 per sample. Data analyses were done by R version 4.2.1 (The R Foundation for Statistical Computing, Vienna, Austria) using open public packages—indicspecies, dplyr, ggpubr, reshape2, tidyverse, and ggplot2, grDevices package were used for visualization.

Pairwise comparisons were evaluated using the Wilcoxon signed-rank test. All pairwise comparisons were calculated between the baseline and the end of the smoothie consumption or WO period. The Wilcoxon rank-sum test was used to evaluate differences in consumption of macronutrients and abundances of bacterial genera between LF and HF intake groups.

Statistical analysis of general health parameters was performed using the SPSS for Windows version 20.0 (SPSS Inc. Chicago, IL, USA). Nonparametric Wilcoxon or Mann–Whitney U-tests were used to evaluate the difference between the medians and distributions of the outcome parameters for the subgroups. Statistical significance was defined at *P* < 0.05.

## Results

### The effects of short-term smoothie consumption on gut microbiota are temporary

**Figure 2.**
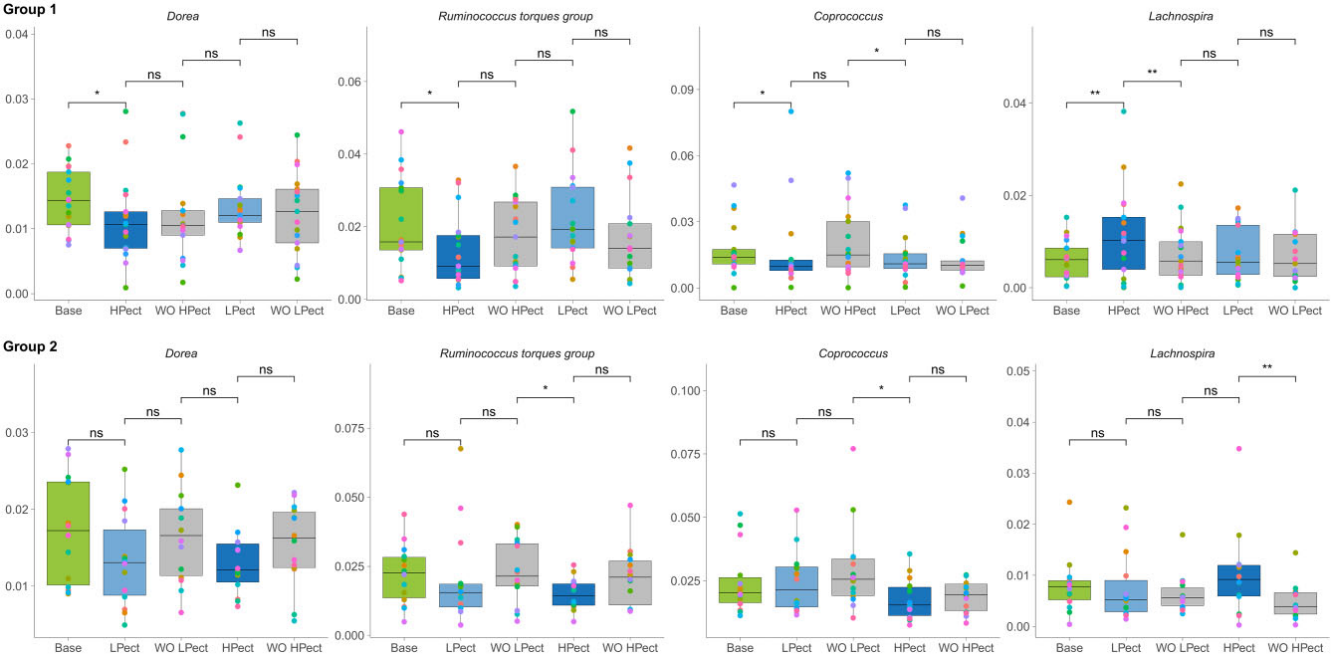
Bacterial up- and down-shifts after smoothie periods and recoveries of abundances after WO periods. Box plots are arranged in period order. Base period characterizes microbiota before the intervention. During the intervention, group 1 consumed first pectin-enriched smoothie (HPect), followed by washout (WO HPect), and consumption of control smoothie (LPect) with washout (WO LPect). In the group 2 intervention was started with the LPect smoothie. Each dot represents one individual, and the same color was used for one person in different periods in the group. *P ≤ 0.05, **P ≤ 0.01, ns—not significant. Y-axis—abundance, normalized to 1.

To exclude the influence of seasonal eating habits on the effect of target smoothie consumption, we used the crossover study design, dividing participants into two groups based on the order of smoothies intake (Fig. 1). We first examined the changes in the abundance of 177 bacterial genera represented in the study group during each of the five periods. We compared samples after the smoothie consumption periods with samples from the pre-smoothie consumption periods. The analysis confirmed that microbiota changes during the first smoothie period did not affect the results of the second smoothie period. Most of the effects caused by smoothie consumption were provisional and did not show differences according to the order of smoothie consumption among the prevalent taxa (Fig. 2). The abundance of *Dorea* decreased after HPect period in group 1, a similar tendency was observed in group 2, but not statistically significantly. The abundances of *R. torques group* and *Coprococcus* decreased after HPect period in both groups. Furthermore, LPect smoothie decreased the level of *Coprococcus* in group 1. The HPect had a remarkable positive effect on the proportion of *Lachnospira* in group 1, and a slight but not significant increase was detected in group 2 (Fig. 2). By the end of the WO periods, the abundances of all these genera returned to the base levels, confirming that the applied crossover method is suitable to evaluate the effect of fiber-enriched foods on the gut microbiota.

### The characteristics of two different fiber intake groups during regular diet period

Consumption of macronutrients based on food records during a regular diet was analyzed in two fiber intake groups. During the study, we formed two focus groups based on the received data. Despite both groups having a low consumption of fruits and vegetables based on the food frequency questionnaire prior to the study, 9 participants had sufficient fiber consumption during the smoothie trial. The LF group consumed fewer fruits and vegetables, as well as other HF foods like whole grains, legumes, and nuts. In contrast, the HF group mainly obtained their sufficient daily fiber intake from whole grain products, legumes, and nuts, with a lesser contribution from fruits and vegetables. While the HF group served as a control, the LF consumption group had more participants as it was the main point of interest. In comparison to fiber intake, the other macro-nutritional values in the daily menu, such as energy, carbohydrates, fat, and protein consumption did not show any significant difference between the two groups (Table 1). However, it is worth noting that fat consumption for both groups was higher than recommended in Estonian nutrition guidelines (Pitsi et al. 2017). Thus, 40% and 36% of the daily consumed energy come from fat in the LF and HF intake groups, respectively.

**Table 1.** The consumption of macronutrients during a regular diet.

Nutrients	LF intake <23 g per day	HF intake ≥23 g per day
Energy (kcal/day)	1768.3 (±281.8)	1827.4 (±147.2)
Fiber (g/day)*	18.9 (±2.8)	27.4 (±4.9)
Carbohydrates (g/day)/(%)	194.1 (±29.1)/44	212.5 (±21.5)/47
Fat (g/day)/(%)	79.5 (±18.5)/40	73.6 (±13.5)/36
Protein (g/day)/(%)	72.1 (±15.9)/16	90.4 (±31.5)/20

Data are presented as average (±stdev). Macronutrients are presented as a percentage from daily energy expenditure of regular diet periods (no smoothie consumption). Distribution was compared between the groups using the Wilcoxon rank-sum test. Statistically significant differences (*P* < 0.05) are shown by asterisk (*). LF—low-fiber intake group (*n* = 22); HF—high-fiber intake group (*n* = 9).

**Figure 3.**
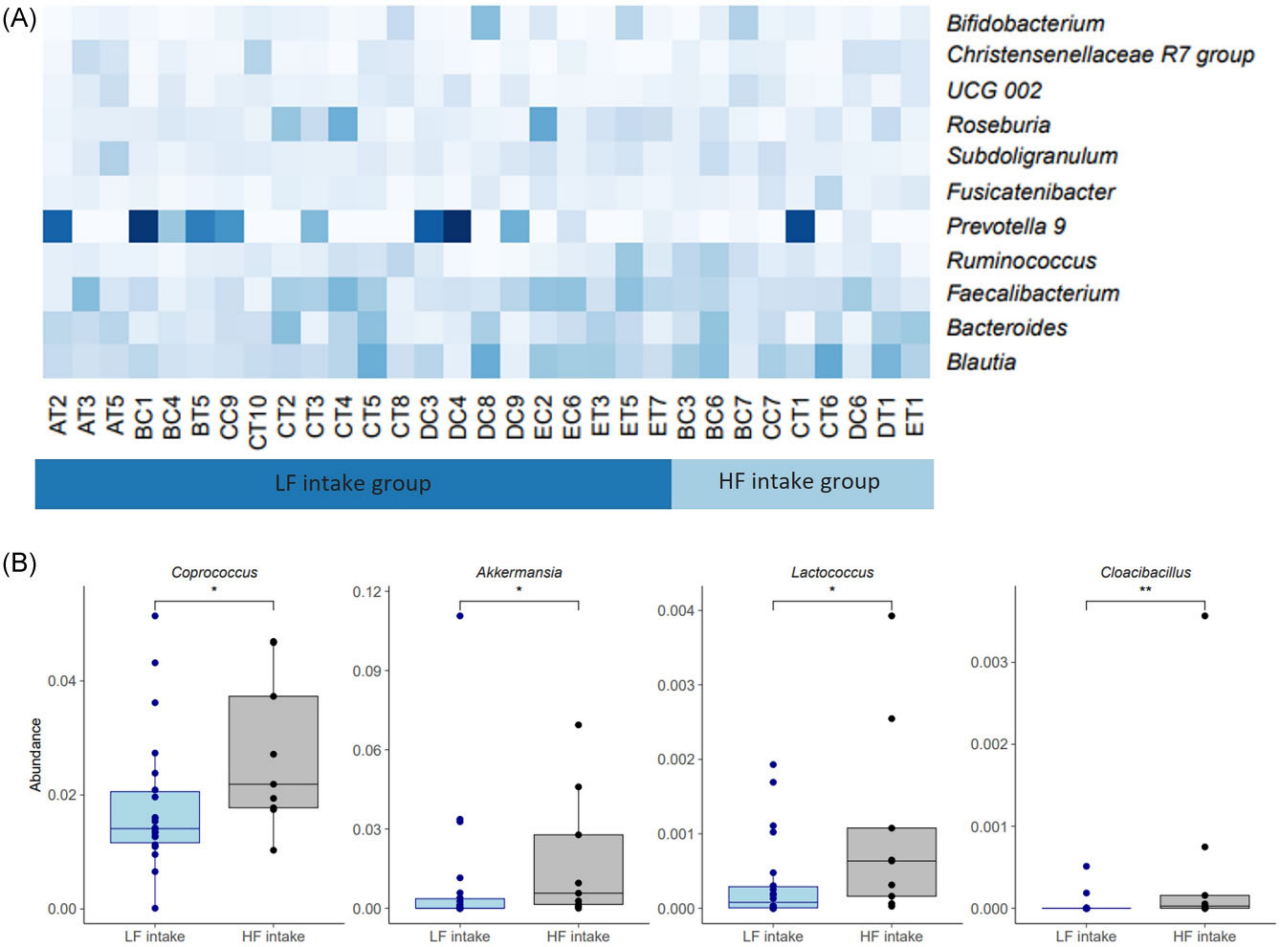
(A) Heatmap illustrating the abundances of the most abundant genera in the base period of LF and HF intake group members, based on the daily fiber intake. (B) At lower levels of abundance, four genera show the differences between LF and HF intake groups in the base period. Dots represent the individuals in the group. Wilcoxon rank-sum tests between LF and HF intake groups were performed (*P ≤ 0.05, **P ≤ 0.01), Y-axis—abundance, normalized to 1. LF—low-fiber; HF—high-fiber.

To investigate the differences in gut microbiota between these two groups, we compared the ten most abundant bacteria genera in the LF and HF intake groups at the beginning of the study. We found that the groups had quite similar gut microbiota composition at the genus level. However, Prevotella-9 dominated over Bacteroides in the LF intake group, whereas the opposite trend was observed in the HF intake group (41% and 22% of the participants had higher level of Prevotella-9 than Bacteroides in the LF and HF intake groups, respectively) (Fig. 3A).

At a lower abundance level, certain bacterial genera exhibit statistically significant differences between LF and HF intake groups (Fig. 3B). We observed that participants who consume <23 g of DF per day (LF intake group) exhibit a decreased abundance of *Coprococcus, Akkermansia, Lactococcus*, and *Cloacibacillus* as compared to the HF intake group (*P*-values 0.046, 0.036, 0.031, and 0.008, respectively).

### The gut microbiota of LF intake group is more affected by smoothie consumption than HF intake group

**Figure 4.**
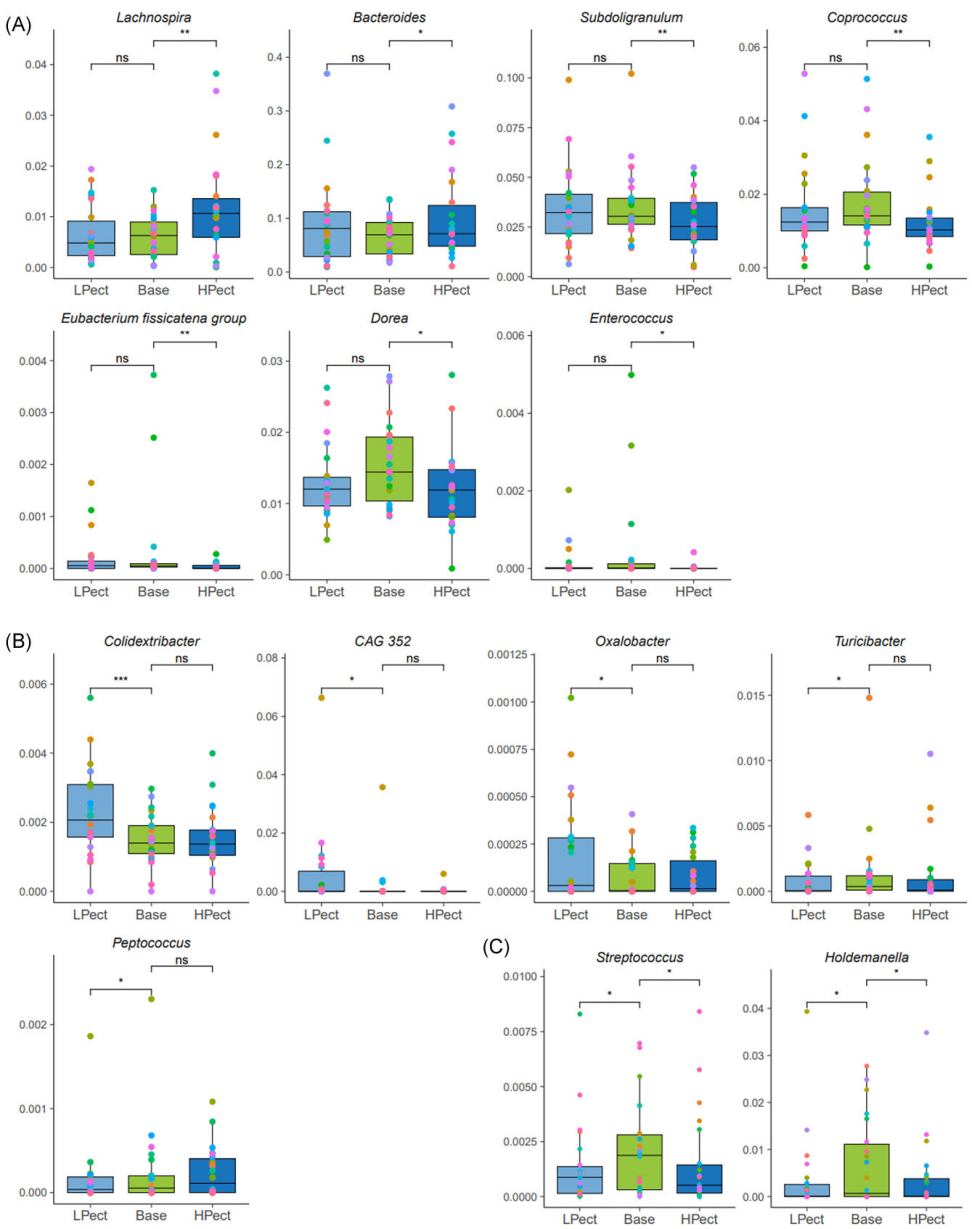
Altered genera in LF intake group. The pairwise comparison was performed using the Wilcoxon signed-rank test for statistical analysis to compare LPect and HPect effects on base period microbiota at the genus level (*P ≤ 0.05, ** *P* ≤ 0.01, *** *P* ≤ 0.001, ns—not significant). Each dot represents one individual, and the same color was used for one person in different periods in the group. Y-axis—abundance, normalized to 1. (A) HPect altered genera; (B) LPect altered genera; (C) Genera altered by both smoothies. LPect—low pectin smoothie; HPect—high pectin smoothie.

To understand how short-term consumption of regular and pectin-enriched fruit smoothies can affect the human microbiota, we analyzed samples from two groups with varying basal fiber intake, as described in Table 1. Regarding the HPect, the abundances of *Lachnospira* and *Bacteroides* increased, whereas the abundances of *Subdoligranulum, Coprococcus, E. fissicatena* group*, Dorea, Lactococcus, Evtepia*, and *Enterococcus* decreased after consumption (Fig. 4A, Supplementary Table S3 and S4). It is worth noting that the baseline abundances of *Coprococcus* and *Lactococcus* were higher for the HF intake group (Fig. 3B). In the LF intake group, the most significant increase in prevalence was detected for *Coldextribacter* after the LPect period. The higher abundance of *CAG-352* and *Oxalobacter* was also observed in the LF intake group post-LPect consumption. On the other hand, the abundances of *Turicibacter, Peptococcus, Defluviitaleaceae_UCG-011, UCG-009*, and *Merdibacter* were reduced after LPect consumption in this group (Fig. 4B, Supplementary Table S3 and S4). Furthermore, the prevalence of *Streptococcus* and *Holdemanella* decreased after both smoothie consumption periods (Fig. 4C, Supplementary Table S3 and S4).

**Figure 5.**
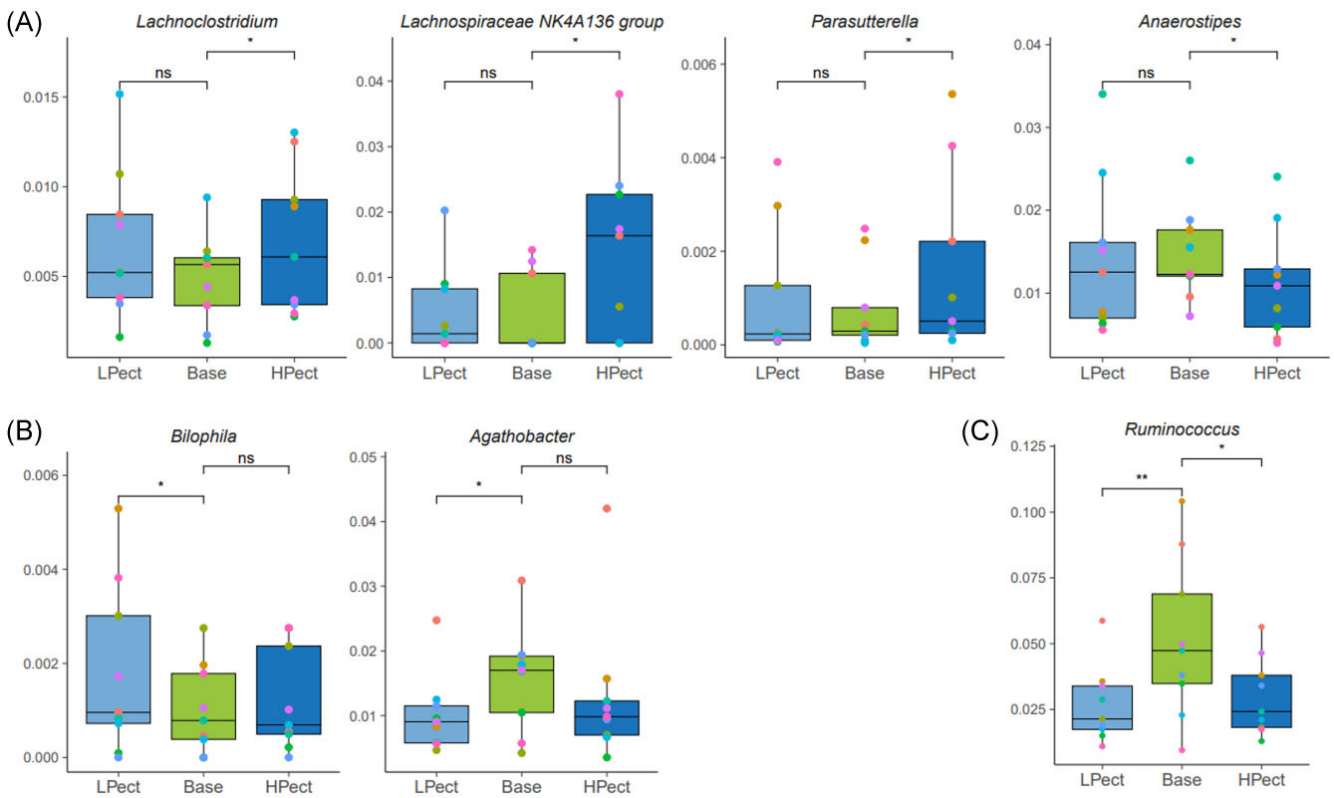
Altered genera in the HF intake group. The pairwise comparison was performed using the Wilcoxon signed-rank test for statistical analysis to compare LPect and HPect effects on base period microbiota at the genus level (*P ≤ 0.05, **P ≤ 0.01, ns—not significant). Each dot represents one individual, and the same color was used for one person in different periods in the group. Y-axis—abundance, normalized to 1. (A) HPect altered genera, (B) LPect altered genera, and (C) Genera altered by both smoothies. LPect—low pectin smoothie; HPect—high pectin smoothie.

In the HF intake group, there were significantly fewer changes after the smoothie periods compared to the LF intake group. In this group, the abundances of *Lachnoclostridium, Lachnospiraceae_NK4A136_group*, and *Parasutterella* were increased, and *Anaersotipes* level reduced after HPect consumption (Fig. 5A). LPect smoothie increased the abundance of *Bilophila* and decreased the proportion of *Agathobacter* (Fig. 5B). Lower levels of *Ruminococcus* were detected after both smoothie periods in the HF intake group, but no significant shifts in the abundances of these genera were observed in the LF intake group (Fig. 5C, Supplementary Table S3 and S4).

**Figure 6.**
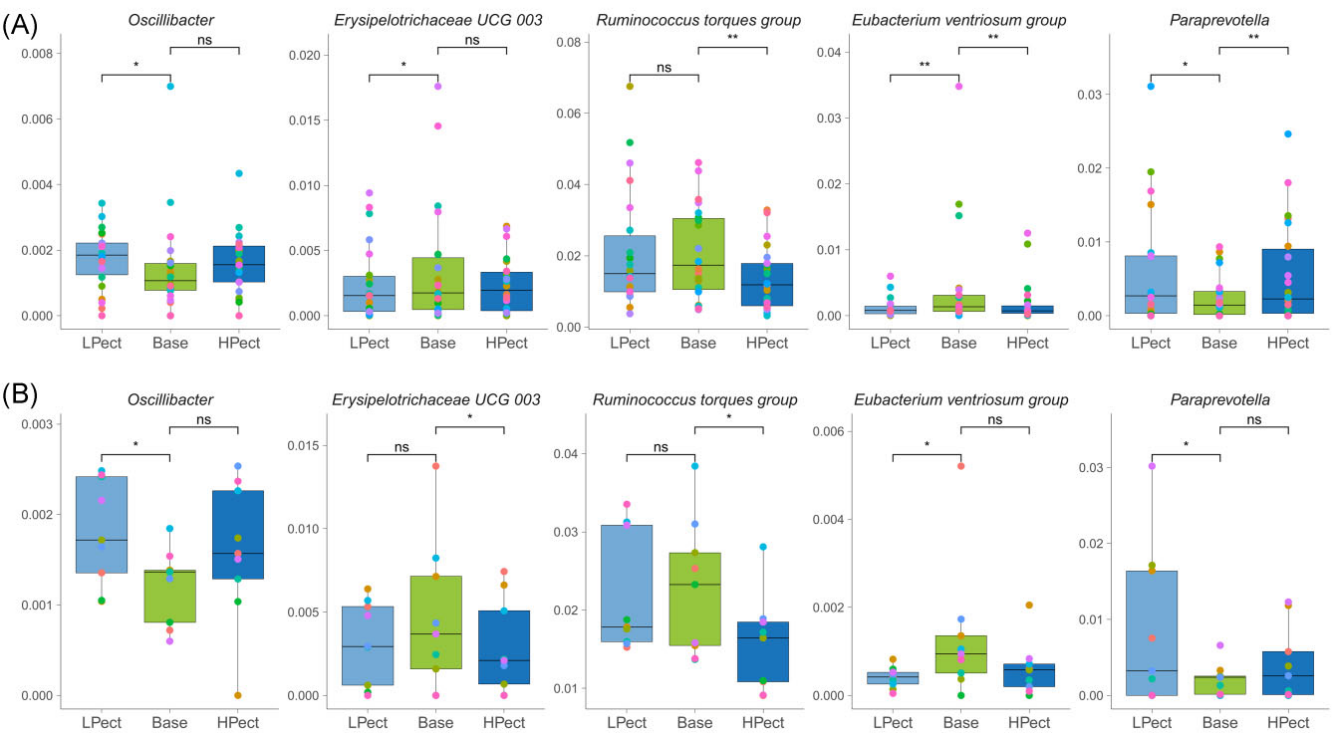
Altered genera in both groups. The pairwise comparison was performed using the Wilcoxon signed-rank test for statistical analysis to compare LPect and HPect effects on base period microbiota at the genus level (*P ≤ 0.05, **P ≤ 0.01, ns—not significant). Each dot represents one individual, and the same color was used for one person in different periods in the group. Y-axis—abundance, normalized to 1. (A) LF intake group; (B) HF intake group. LPect—low pectin smoothie; HPect—high pectin smoothie.

There were several genera that exhibited significant changes in both groups after the consumption of smoothie. The abundance of *Oscillibacter* increased after the LPect period in both groups. The prevalence of *Erysipelotrichaceae_UCG-003* decreased after LPect consumption in the LF intake group and after the HPect period in the HF intake group. The abundance of *R. torques group* decreased after the HPect period in both groups. The level of *E. ventriosum group* decreased following both smoothie periods in the LF intake group and after LPect consumption in the HF intake group. Finally, the abundance of *Paraprevotella* increased after both smoothie consumption periods in the LF intake group and after LPect in the HF intake group. (Fig. 6, Supplementary Table S3 and S4).

### HF intake is associated with lower body weight and better health parameters

The comparative health indicators of the LF and HF intake study groups during the base period are presented in Supplementary Table S5. The median BMI of subjects in the HF intake group was statistically significantly lower compared to the median BMI of the LF intake group (20.6 vs. 23.0, Mann–Whitney U test, *P* = 0.048). In addition, compared to the LF intake group, the HF intake group showed slightly lower mean blood glucose (5.1 vs. 4.8 mmol/l, Mann–Whitney U test, *P* = 0.048) and hemoglobin levels (134.5 vs. 130 g/l, Mann–Whitney U test, *P* = 0.048). Because men have a higher range of reference values for uric acid and hemoglobin, these data were analyzed separately for women. By excluding three men from the analysis, the statistical difference in hemoglobin levels between groups disappeared. However, a statistically significantly higher level of uric acid was revealed in the LF intake group (*n* = 20) compared to the HF intake group (*n* = 8) (261.0 vs. 229.5 µmol/l, Mann–Whitney U test, *P* = 0.03). No differences were found between other health parameters in the baseline period. Baseline differences were maintained throughout the study periods. No significant changes in health parameters were obtained with the use of smoothies in the LF intake group except for glucose, where the between-group difference disappeared by the end of the study.

## Discussion

Participants whose everyday fiber intake was lower than 23 g per day had much more altered gut microbiota genera in response to increased fiber intake. Moreover, these changes are directed and encompass distinct genera. It has been established that a person’s dietary habits can influence the changes of gut microbiota, and whether they respond to a particular treatment or not (Rose et al. 2015, Tap et al. 2015, Procházková et al. 2023). In this study, we showed that the effects of regular and DF-enriched fruit smoothies depend on the baseline consumption of fiber. Thus, we may expect some substantial positive effect of DF on gut microbiota in case of low baseline fiber consumption. However, these changes are short-term and disappear after restriction of increased fiber intake.

The most abundant bacteria did not show significant changes between these groups; however, the ratio of the abundances of *Prevotella 9* and *Bacteroides* seems to be different (41% and 22% of the participants have a higher abundance of *Prevotella 9* than *Bacteroides* in LF intake and HF intake group, respectively). Previously, it has been known that *Prevotella* should dominate for people who consume more vegetarian food rich in DF, and *Bacteroides* is more common for people who prefer animal-based food rich in fat and protein (Wu et al. 2011, Roager et al. 2014). Differing results in our study may be caused by the fact that the landscape of *Prevotella* impact on gut microbiota is diverse and cannot be associated with increased fiber consumption only. For instance, healthy subjects who exhibited improved glucose metabolism after kernel-based bread intake had a higher *Prevotella/Bacteroides* ratio (Kovatcheva-Datchary et al. 2015) or lost more body weight and fat compared to individuals with low *Prevotella/Bacteroides* in response to a diet rich in fiber (Hjorth et al. 2019). Also, a habitual diet rich in fibers can promote the growth of different *Prevotella* strains (De Filippis et al. 2019), some of which do not have a positive impact on human health, and their increased levels could be linked with inflammation (Larsen 2017). It is important to mention that both *Prevotella* and *Bacteroides* are the primary pectin consumers and could equally benefit from a HF smoothie (Larsen et al. 2019).

On the other hand, we observed distinctive changes in the proportion of four genera between groups consuming various amounts of fiber. Participants who consumed <23 g of DF per day exhibited a decreased abundance of *Coprococcus, Akkermansia, Lactococcus*, and *Cloacibacillus* as compared to the HF intake group. It was shown that increased levels of these bacterial genera in the gut relate to better health parameters and digestive activity. Some species from the genus *Coprococcus* are butyrate producers and thereby could have a supportive effect on human health and inhibit inflammatory processes in the colon (Rivière et al. 2016). *Coprococcus* can also participate in gut-brain communications and has a positive correlation with a better mental and emotional state (Valles-Colomer et al. 2019). There is some evidence that shows an association of *Coprococcus comes* and higher BMI (Liu et al. 2017, Adamberg et al. 2020). Conversely, in our study, the HF intake group had a higher *Coprococcus* and a lower BMI than that in LF intake group. However, it can be explained by the different species compositions of coprococci that we could not discriminate this time. Also, the abundance of *Akkermansia* was higher in the HF intake group. Most of the cases, *Akkermansia*, especially *Akkermansia muciniphila* is known as a good health marker due to the ability to renew the mucus layer and thereby have a supportive effect on maintaining the intestinal integrity. Many diseases are associated with a decreased level or absence of *Akkermansia* (Geerlings et al. 2018). At the same time, we have to pay attention that a high level of the abundance of *Akkermansia* is associated with constipation and longer gut transit time (Vandeputte et al. 2016, Asnicar et al. 2021). Since a sedentary lifestyle and a low Bristol score were inclusion criteria for the study, this may explain why some participants have very high levels of *Akkermansia*.

We found that the abundance of *Lachnospira* increased after HPect consumption in LF intake group, while the abundance remained unchanged in HF intake group. It could be assumed that the effect we observed after HPect period is associated with pectin digestion. HPect contains a notable amount of pectin and individuals who consume a lot of fruits have more *Lachnospira* in their gut (Kable et al. 2022). *Lachnospira* can use pectin as a substrate for acetate production (Bang et al. 2018, Beukema et al. 2020) and could contribute to the formation of acidic environment in the colon, which in turn impedes the growth of pathogenic bacteria. Moreover, the genus *Lachnospira* has a higher abundance among lean people, and a negative correlation with total and LDL cholesterol levels is described (Companys et al. 2021).

One more positive effect of increased fiber consumption was the reduction of mucus-degrading *R. torques group* after HPect intake for both groups. The proportion of this bacterium is usually higher for individuals with intestinal inflammation. It has been shown that the mucolytic *R. torques* is markedly increased in noninflamed UC (ulcerative colitis) and Crohn’s disease epithelium (Png et al. 2010). Therefore, we should note that, besides considering the daily quantity of DF consumption, it is essential to pay attention to the type of consumed DF.

Regarding the health risks, we confirmed an already known association of higher fiber intake with lower BMI (Hadrévi et al. 2017, Ito et al. 2023). In addition, the study shows that women in the LF intake group have higher uric acid levels compared to the HF intake group. LF Western diet is associated with higher levels of uric acid, which increases the risk of gout. The association of a HF diet with lower uric acid levels in women has also been shown previously (Chen et al. 2020). Although we observed between-group differences in BMI and health parameters at baseline, short-term increased fiber intake was not sufficient to improve health outcomes in either study group.

It is worth mentioning that people tend to appreciate their eating habits not very adequately, which limited the study by decreasing the number of recruited participants based on the questionnaire. Despite not having healthy food habits, according to the questionnaire, nearly a third of the study participants were in good health.

A weakness of the study is that few men participated, and men also dropped out more easily, resulting in a biased sample towards women. Another important aspect to consider is that the small group is susceptible to individual variability, and each sample can significantly impact the overall results.

## Conclusion

In our study, we demonstrated that industrial by-products such as apple pomace could be used as a main component, along with other fibers, to create snacks with health benefits that enrich the regular diet and increase fiber consumption. Using a cross-section study, in which participants filled dietary questionnaires, tested various human health parameters, took blood tests, and determined changes in gut microbiota. We revealed that baseline fiber intake determines the response of gut microbiota to smoothie consumption. Although smoothie consumption did not provide benefits on the blood markers, we observed that LF consumers had more positive changes in their microbiota at the genus level after smoothie intake. Hence, a pectin-enriched smoothie could promote the growth of beneficial gut bacteria. Moreover, we estimated the correlation between food components and definite gut bacteria. However, this effect is transient, as most changes in microbiota recovered during WO time. Certainly, for evaluating the lasting effects on health characteristics (BMI, blood cholesterol etc.), the long-term trials should be carried out.

## Supplementary Material

xtae001_Supplemental_Files

## Data Availability

The data presented in this study is available on SRA database: https://www.ncbi.nlm.nih.gov/sra/PRJNA981491
