# Supplementary material for: Short-term pectin-enriched smoothie consumption has beneficial effects on the gut microbiota of low-fiber consumers
Source: FEMS Microbes. 2024 Feb 2;5:xtae001. doi: 10.1093/femsmc/xtae001 (PMC10880814; doi:10.1093/femsmc/xtae001)
Supplement: xtae001_Supplemental_Files [file xtae001_supplemental_files.zip › Supplementary data.docx]

Supplementary data

**Table S1:** Recipe of the smoothies

| **Component *** | **LPect** | **HPect** |
| --- | --- | --- |
| Banana puree | 19.0 | 8.5 |
| Peach puree | 39.0 | 28.9 |
| Pineapple puree | 28.1 | 15.3 |
| Pineapple concentrate | 0.9 | 0.9 |
| Light grape concentrate | 1.2 | 1.2 |
| Water | 6.8 | 6.8 |
| Apple puree made out of pomace | 0.0 | 36.0 |
| Fiber supplement** | 0.0 | 2.5 |
| Strach | 5.0 | 0.0 |

*All the amounts of ingredients are presented as g per 100 g of product

**Calm your rumbly tummy (Elsavie, Estonia), 100 g of product contains: citrus fiber 29.5 g, psyllium 23.5 g, beta-glucan 9.5 g, Marigold extract 2.0 g, Turmeric 1.0 g, Aloe extract 0.5 g

LPect – low pectin smoothie; HPect – high pectin smoothie

**Table S2:** Nutritional values of smoothies per 100 g

| **Nutrient*** | **LPect** | **HPect** |
| --- | --- | --- |
| Energy, kJ/kcal | 294/69 | 225/60 |
| Fat, g | <0.5 | <0.5 |
| Including saturated fat, g | 0 | 0 |
| Carbohydrate, g | 17.3 | 15.8 |
| Including sugars, g  Including fiber**, g | 11.8  1.2 | 11.3  2.9 |
| Protein, g | 0.7 | 0.7 |
| Salt, g | 0.01 | 0.02 |

*Nutritional values were determined by the Estonian Veterinary and Food Laboratory

**Fiber content determined by methods AOAC 985.29 + AOAC 2001.03 HPLC

LPect – low pectin smoothie; HPect – high pectin smoothie

**Table S3:** Altered genera in response to low (LPect) or high pectin smoothie (HPect) consumption. **A.** LF intake group specific results, **B.** HF intake group specific results, **C.** Shared results of both groups. LF – low fiber; HF- high fiber.

*Median of the abundances <0.0006

Statistically significant results are illustrated as + (positive effect) or – (negative effect), 0 – no statistically significant results. Wilcoxon´s signed-rank test was performed to compare LPect/HPect to base period sample.

**Table S4:** Abundances (normalized to 1) of altered genera in HF- and LF intake groups, data is presented as group median and Q1 and Q3 values. * - means that the median abundance is lower than 0.0006. LF – low fiber, HF – high fiber; LPect – low pectin smoothie; HPect – high pectin smoothie.

**Table S5:** The comparative health indicators of the Low Fiber and High Fiber intake groups

| **Health parameters** | **LF (n = 22)** | **HF (n = 9)** |
| --- | --- | --- |
| Age (years) | 37.5 (29.8-41) | 37.0 (32.5-41) |
| BMI (kg/m^2^)***** | 23.0 (20.3-27.5) | 20.6 (19.1-22.9) |
| Glucose (mmol/L)* | 5.1 (4.8-5.6) | 4.8 (4.6-5.2) |
| Cholesterol (mmol/L) | 4.5 (4.1-5.0) | 4.6 (4.3-5.0) |
| HDL (mmol/L) | 1.6 (1.4-2.3) | 2.0 (1.7-2.3) |
| LDL (mmol/L) | 2.5 (2.2-3.1) | 2.4 (2.1-2.9) |
| non-HDL cholesterol | 2.7 (2.2-3.3) | 2.8 (2.2-3.0) |
| Triglycerides (mmol/L) | 0.8 (0.7-1.1) | 0.9 (0.6-1.1) |
| Uric acid (μmol/L)*^a^ | 262.0 (214.3-308.8) | 235.0 (205.0-251.5) |
| C-reactive protein (mg/L) | 0.1 (0.1-2.0) | 0.1 (0.1-2.5) |
| ASAT (U/L) | 21.0 (17.8-23.3) | 18.0 (15.0-26.0) |
| ALAT (U/L) | 16.5 (12.8-23.5) | 13.0 (12.0-26.0) |
| GGT (U/L) | 18.5 (13.8-23) | 16.0 (13.0-22.0) |
| WBC (E9/L) | 5.5 (4.9-6.7) | 5.7 (5.0-6.5) |
| RBC (E12/L) | 4.6 (4.3-4.8) | 4.4 (4.2-4.6) |
| Hemoglobin (g/L)* | 134.5 (130.5-140.5) | 130.0 (127.5-132.0) |

Data is presented as median (25–75 *%*). Distribution of parameters was compared between the subgroups using the Mann-Whitney U test. Statistically significant differences (p<0.05) are shown by asterisk (*).

^a^ These data were analyzed separately for women: LF group (n = 20), HF group (n = 8)

LF – low fiber; HF – high fiber
