# Supplementary material for: Short-term pectin-enriched smoothie consumption has beneficial effects on the gut microbiota of low-fiber consumers
Source: FEMS Microbes. 2024 Feb 2;5:xtae001. doi: 10.1093/femsmc/xtae001 (PMC10880814; doi:10.1093/femsmc/xtae001)
Supplement: xtae001_Supplemental_Files [file xtae001_supplemental_files.zip › FEMSMC-2023-024 supplementary data - reviews and author response.docx]

**Reviews of manuscript FEMSMC-2023-024**

**Reviewer 1**

**Recommendation: Reject**

Susan Pihelgas et al. present data about the consumption of pectin-enriched smoothies in two groups of subjects, low fiber and high fiber intake, but several key aspects prevent me from suggesting this article for publication.

General comments

In the title, I think it is more accurate to say apple-enriched.

Add at least one line somewhere about the microbial contribution of apples in the smoothies (https://doi.org/10.3389/fmicb.2019.01629).

I could not find the proportion of women in each subgroup, except in Table S5 where it says that some data was analyzed separately, apparently most subjects were women (91% and 89% in each subgroup, respectively). If so, it is very important to mention this and it is also a weakness, especially because I see no reason why mostly women would be eager to participate, UNLESS they were having some GI issues and perhaps believed that participating would somehow help them? Or is it that males have less time available for participating in such studies?

Recruitment

Why exactly did you ask potential subjects to have a modest consumption of fruits and vegetables? If this paper is part of a bigger study, it is important to acknowledge. How did you control or make sure that the subjects actually consumed the smoothies?

DNA extraction and sequencing

How exactly does the swab work (I guess you use it to sample the fecal material directly?) and how much fecal material did you use?

RESULTS

The word “provisional” sound strange, is there not any other word to say temporary?

Table 1

I am concerned about the difference in the number of subjects in each group. Could you please defend your decision? Why not balancing the number of subjects, for example below and above the average fiber intake? This is the weakest aspect of this paper.

Discussion

The first statement may be correct, but it does not sound accurate based on current literature on gut microbial ecology. Baseline parameters influence most of the response to any dietary intervention. I would dig more into the literature and use some references (examples: doi: 10.1080/10643389.2014.1000761; doi: 10.1016/j.cell.2016.10.043) to rephrase this.

Conclusion

Line 323. What about other ingredients such as pectin, psyllium, and beta-glucan fibers? In other words, if you did not use pomace alone, how can you be sure that the snacks were “healthy” because of the pomace?

Table S5. I think it is glucose instead of “glycose”.

**Reviewer 2**

**Recommendation: Minor Revision**

The authors provide an assessment of gut microbiota and some peripheral parameters before and after study participants consumed drinks of varied fiber content. The study seems to be well thought out, with washout periods before and after high/low fiber intake to investigate the persistence of microbiota changes.

While thorough in their analysis, some of the data presentation could be improved. The volume of graphs shown in Figures 2 and 4-6 should be reduced. Either find a way to incorporate multiple bacteria in one panel, or show representative data (the remainder could be supplemental). As presented, it is a little overwhelming for the reader.

In the assessment of the most abundant genera in figure 3, how did the genera called out in other figures relate in terms of abundance?

While the vast majority of comparisons show no difference between groups, the effect of outliers needs to be assessed/accounted for in some of the groups that do have stated significant differences. Main examples seem to be Cloacibacillus in fig 3B, Enterococcus and Fissicatina group in fig 4. There are several others that seem to have a very wide distribution of data points while maintaining significance.

It is acknowledged that minority genera do play a part in the microbiota, some of the highlighted groups have vanishingly low abundance, and in absolute terms equally low difference between treatment groups. Do the authors think these are biologically relevant?

I assume the colours of the data points represent individual participants tracked through each particular analysis? Either way, this should be clarified in the figure legends.

Other specific points

• The headers on revised figure panels need to be tidied up with regard to italicizing of genera names and removing “.” And “_” characters from the group names that come out of the SILVA alignment

• Specific R packages used should be noted and where appropriate, cited.

• Remove surplus apostrophe in header of section 3.3

**Author's Response to Decision Letter for (FEMSMC-2023-024)**

Dear Editor and Reviewers,

Thank you for your valuable feedback and comments. They have greatly improved the quality of the manuscript. Please find our detailed response to the reviewers below:

Reviewer: 1

Comments to the Author

Susan Pihelgas et al. present data about the consumption of pectin-enriched smoothies in two groups of subjects, low fiber, and high fiber intake, but several key aspects prevent me from suggesting this article for publication.

General comments

1. In the title, I think it is more accurate to say apple-enriched. Add at least one line somewhere about the microbial contribution of apples in the smoothies (https://doi.org/10.3389/fmicb.2019.01629).

Answer: The main aim of the project we followed in our nutritional study was to valorize the apple pomace. To achieve this, we developed a smoothie that contained an increased amount of apple pomace. Additionally, the smoothie was enriched with soluble fibers (2.5 g/100 g), which contained 45 % citrus fiber, the main component of which is pectin. The composition of it is detailed in Table S1. We then conducted a crossover study where participants consumed both control and apple pomace-enriched smoothies. Based on the data, it is incorrect to say that apple puree was the dominant ingredient, as peach was also present in a high amount along with other fiber-rich fruits (52.7 g/100 g). It is important to note that the main fiber in pomace is pectin (5-17% of dry mass, https://doi.org/10.1590/S0104-14282005000200012). Therefore, it may be misleading to use the term "apple-enriched" in the title as the main component in the fiber-enriched smoothie was a mixture of pectins.

2. I could not find the proportion of women in each subgroup, except in Table S5 where it says that some data was analyzed separately, apparently most subjects were women (91% and 89% in each subgroup, respectively). If so, it is very important to mention this and it is also a weakness, especially because I see no reason why mostly women would be eager to participate, UNLESS they were having some GI issues and perhaps believed that participating would somehow help them? Or is it that males have less time available for participating in such studies?

Answer: We acknowledge that the high number of women in this study is a limitation. We tried to encourage more men to participate, but it was difficult due to several reasons. One of the reasons for the low participation of men could be that they are not the primary consumers of smoothies. Moreover, men dropped out of the study more frequently due to its length and associated obligations, leading to a biased sample towards women. However, it is important to note that this is a common tendency in this type of research, where women tend to be more active participants. The main criteria for participation were a sedentary lifestyle, low consumption of fruits and vegetables, and constipation, which are not gender specific. Although this may introduce some biases to the conclusions, we hope that the findings of this study can be generalized to the entire population or applied to targeted smoothie consumers.

The necessary additions concerning the selection of the sample have been relocated to the text for better clarity in paragraph 2.2 Recruitment of study participants and design of the study (lines 94-96).

Out of 368 volunteers who completed the questionnaire, 55 were men, representing 15% of the total participants. Out of the 39 eligible participants, 31 completed the study, of which 3 (9.7%) were men.

3. Recruitment. Why exactly did you ask potential subjects to have a modest consumption of fruits and vegetables? If this paper is part of a bigger study, it is important to acknowledge. How did you control or make sure that the subjects actually consumed the smoothies?

Answer: This study was an independent investigation focused on examining the impact of pectin fibers on the human health parameters, gut microbiota in particular. Since people generally consume insufficient amounts of fiber, the primary inclusion criteria for the study were individuals with low consumption of fruits and vegetables, to evaluate the effect of increased fiber intake on gut microbiota. It is important to note that consuming large amounts of pectin-enriched smoothies daily can affect the nutrient balance in individuals who already consume adequate amounts of dietary fibers. This could lead to an imbalance of other important nutrients, which we wanted to avoid.

To monitor the participants' diet, we requested them to maintain food diaries, which we examined for both the base period and the study period. The use of food diaries is mentioned in section 2.3. Data collection.

For clarity, a reference to using a food diary has been added to the sentence below (lines 105-107).

For data analysis, subjects were grouped into LF (low-fiber), and HF (high-fiber) intake cohorts based on the analysis of food diaries according to the Estonian nutrition and movement recommendations (Pitsi et al., 2017) and their everyday dietary fiber consumption (during base and washout periods).

For clarity the sentence below has been added to 2.3. Data collection (lines 115-116)

The participants were asked to fill out the exact amount of smoothies they consumed.

4. DNA extraction and sequencing. How exactly does the swab work (I guess you use it to sample the fecal material directly?) and how much fecal material did you use?

Answer: Participants were instructed to collect fecal samples at their homes using a FecesCatcher (https://www.youtube.com/watch?v=hsAyURaBKv0). The process involved collecting feces with the FecesCatcher and then taking a sample with a swab. The swab should be pulled into the feces in three different places and then placed into a tube containing an inactivation solution. The amount of feces collected was sufficient when the solution became cloudy. For analysis, the extracted DNA concentration was normalized, not the amount of feces collected.

5. RESULTS. The word “provisional” sound strange, is there not any other word to say temporary?

Answer: Changed in text: 3.1. (line 165)

3.1. The effects of short-term smoothie consumption on gut microbiota are temporary

6. Table 1. I am concerned about the difference in the number of subjects in each group. Could you please defend your decision? Why not balancing the number of subjects, for example below and above the average fiber intake? This is the weakest aspect of this paper.

Answer: Our study focused on individuals who had a low daily intake of fruits and vegetables. During the study, we formed two focus groups based on the received data. Despite both groups having a low consumption of fruits and vegetables based on the food frequency questionnaire prior to the study, 9 participants had sufficient fiber consumption during the smoothie trial. The low-fiber group consumed fewer fruits and vegetables, as well as other high-fiber foods like whole grains, legumes, and nuts. In contrast, the high-fiber group mainly obtained their sufficient daily fiber intake from whole grain products, legumes, and nuts, with a lesser contribution from fruits and vegetables. While the high-fiber group served as a control, the low-fiber consumption group had more participants as it was the main point of interest.

To differentiate between the two groups based on fiber intake during the regular diet period, the following text has been added to section 3.2. (lines 184-191).

During the study, we formed two focus groups based on the received data. Despite both groups having a low consumption of fruits and vegetables based on the food frequency questionnaire prior to the study, 9 participants had sufficient fiber consumption during the smoothie trial. The low-fiber group consumed fewer fruits and vegetables, as well as other high-fiber foods like whole grains, legumes, and nuts. In contrast, the high-fiber group mainly obtained their sufficient daily fiber intake from whole grain products, legumes, and nuts, with a lesser contribution from fruits and vegetables. While the high-fiber group served as a control, the low-fiber consumption group had more participants as it was the main point of interest.

7. Discussion. The first statement may be correct, but it does not sound accurate based on current literature on gut microbial ecology. Baseline parameters influence most of the response to any dietary intervention. I would dig more into the literature and use some references (examples: doi: 10.1080/10643389.2014.1000761; doi: 10.1016/j.cell.2016.10.043) to rephrase this.

Answer: Our major conclusion stated at the beginning of the discussion is that the effects of regular and dietary fiber-enriched fruit smoothies depend on the baseline consumption of fiber.

Based on the feedback provided, the discussion section has been revised to specify the statement mentioned (lines 264-267).

It has been established that a person's dietary habits can influence the changes of gut microbiota, and whether they respond to a particular treatment or not (Procházková et al 2023, Tap et al 2015, Rose et al 2015). In this study, we showed that the effects of regular and dietary fiber-enriched fruit smoothies depend on the baseline consumption of fiber.

8. Conclusion. Line 323. What about other ingredients such as pectin, psyllium, and beta-glucan fibers? In other words, if you did not use pomace alone, how can you be sure that the snacks were “healthy” because of the pomace?

Answer: Based on the feedback provided, the conclusion section has been revised to correct the statement mentioned (lines 339-341).

In our study, we demonstrated that industrial by-products such as apple pomace could be used as a main component, along with other fibers, to create healthy snacks that enrich the regular diet and increase fiber consumption.

9. Table S5. I think it is glucose instead of “glycose”.

Answer: Changed in text: Table S5

Reviewer: 2

Comments to the Author

The authors provide an assessment of gut microbiota and some peripheral parameters before and after study participants consumed drinks of varied fiber content. The study seems to be well thought out, with washout periods before and after high/low fiber intake to investigate the persistence of microbiota changes.

1. While thorough in their analysis, some of the data presentation could be improved. The volume of graphs shown in Figures 2 and 4-6 should be reduced. Either find a way to incorporate multiple bacteria in one panel, or show representative data (the remainder could be supplemental). As presented, it is a little overwhelming for the reader.

Answer: During our data analysis and preparation for publication, we experimented with various visualization options. After careful consideration, we chose the ones that we believed were the most effective. We included representative data in the main text and added the whole cohort to the supplementary data based on statistical significance and evaluation. In the main text, we only present the most important findings of the study. We did not want to show any bias towards one bacterial genus over another based solely on its abundance. Bacterial genera with low abundance may have significant functions in gut microbiota, and we should not underestimate their importance. However, we have reduced the graphs as much as possible to maintain readability, as recommended.

Updated versions of figures are uploaded:

2. In the assessment of the most abundant genera in figure 3, how did the genera called out in other figures relate in terms of abundance?

Answer: Figure 3 represents the ten most abundant genera of base period samples for both groups. In other figures, we only present the results that show statistically significant changes between groups, which were not necessarily dominant before the study began. For example, the abundance of Bacteroides increased and Subdoligranulum decreased after consuming HPect smoothie in the LF intake group (Fig. 4A), and Ruminococcus decreased after both smoothies’ consumption in HF intake group (Fig. 5C). Other dominant bacteria did not show any significant changes in the response to increased fiber intake.

3. While the vast majority of comparisons show no difference between groups, the effect of outliers needs to be assessed/accounted for in some of the groups that do have stated significant differences. Main examples seem to be Cloacibacillus in fig 3B, Enterococcus and Fissicatina group in fig 4. There are several others that seem to have a very wide distribution of data points while maintaining significance.

Answer: In our study, the HF intake group was relatively small (n=9). To analyze the data, we used the ggpubr package and the stat_compare_means function in R 4.2.1. When we excluded the outliers, we found that another outlier appeared. For instance, if we removed the sample with the highest abundance of Cloacibacillus from the analysis, the statistically significant difference remained, but another outlier emerged.

It's important to consider that the small group is susceptible to individual variability, and each sample can significantly impact the overall results. For instance, in Figure 4, we compared the samples taken before and after consuming LPect and HPect smoothies for each person in the group separately and in this case every change does matter and has some impact.

4. It is acknowledged that minority genera do play a part in the microbiota, some of the highlighted groups have vanishingly low abundance, and in absolute terms equally low difference between treatment groups. Do the authors think these are biologically relevant?

Answer: Although some detected genera have very low abundance, it is important to consider that microbiota diversity is a crucial aspect in maintaining good health conditions and even minor bacteria species could be important regarding the whole consortia and impact for the gut health. For example, it was shown that low abundance species play an essential role in immunogenicity (doi: 10.1080/19490976.2022.2104086) or contribute to dysbiosis (https://doi.org/10.3389/fcimb.2021.689197). To analyze the data accurately, minimal read count was taken into account to set the abundance threshold to at least 0.0006 to minimize the effect of noise and remove these data from analysis.

5. I assume the colors of the data points represent individual participants tracked through each particular analysis? Either way, this should be clarified in the figure legends.

Answer: Yes, you are right. Each dot represents one color-coded individual across different time periods. Additional information is provided in the legends of figures 2-6 to emphasize this.

6. Other specific points

• The headers on revised figure panels need to be tidied up with regard to italicizing of genera names and removing “.” And “_” characters from the group names that come out of the SILVA alignment

Answer: Requested changes are made (figures 2-6).

• Specific R packages used should be noted and where appropriate, cited.

Answer: The following information is added (lines 154-155): Data analysis was done by R version 4.2.1 (The R Foundation for Statistical Computing, Vienna, Austria) using open-source packages including indicspecies, dplyr, ggpubr, reshape2, tidyverse and ggplot2. The grDevices package was used for visualization.

• Remove surplus apostrophe in header of section 3.3

Answer: Change is made (line 214).

**Reviews of manuscript FEMSMC-2023-024.r1**

**Reviewer 2**

**Recommendation: Accept**

In my view, the authors have made a sufficient changes/clarifications to the manuscript to warrant publication.

**Reviewer 3**

**Recommendation: Accept**

The authors have improved and adapted the manuscript from previous versions. I think the last line of the abstract needs modification because you are not testing the methodology of estimating relationships but undertaking a dietary intervention. "Our results showed that the short-term approach is effective in estimating relationships between food components and gut bacteria.
